# iRegNet3D: three-dimensional integrated regulatory network for the genomic analysis of coding and non-coding disease mutations

**DOI:** 10.1186/s13059-016-1138-2

**Published:** 2017-01-18

**Authors:** Siqi Liang, Nathaniel D. Tippens, Yaoda Zhou, Matthew Mort, Peter D. Stenson, David N. Cooper, Haiyuan Yu

**Affiliations:** 1 Department of Biological Statistics and Computational Biology, Cornell University, Ithaca, NY 14853 USA; 2 Weill Institute for Cell and Molecular Biology, Ithaca, NY 14853 USA; 3 Institute of Medical Genetics, Cardiff University, Heath Park, Cardiff, CF14 4XN UK

**Keywords:** iRegNet3D, Transcriptional regulation, TF-DNA interaction network, TF-TF interaction network, Chromatin interaction network, Inherited disease, Disease-associated mutation, Missense mutation, Non-coding mutation

## Abstract

The mechanistic details of most disease-causing mutations remain poorly explored within the context of regulatory networks. We present a high-resolution three-dimensional integrated regulatory network (iRegNet3D) in the form of a web tool, where we resolve the interfaces of all known transcription factor (TF)-TF, TF-DNA and chromatin-chromatin interactions for the analysis of both coding and non-coding disease-associated mutations to obtain mechanistic insights into their functional impact. Using iRegNet3D, we find that disease-associated mutations may perturb the regulatory network through diverse mechanisms including chromatin looping. iRegNet3D promises to be an indispensable tool in large-scale sequencing and disease association studies.

**Electronic supplementary material:**

The online version of this article (doi:10.1186/s13059-016-1138-2) contains supplementary material, which is available to authorized users.

## Background

Genetic factors underlie many human diseases [1] and are being identified at an ever-increasing rate through both targeted and genome-scale sequencing studies. For example, genome-wide association studies (GWASs) have identified more than 20,000 robust genotype-phenotype associations [2, 3]. Recent technological advances, including next-generation sequencing (NGS)-based approaches, have given rise to the discovery of a plethora of disease-associated genes and mutations [4, 5]. Yet little of this abundance of information has been translated into drug development and therapeutic applications, although disease-associated genes and mutations are being identified at an increasingly high rate. Indeed, most US Food and Drug Administration (FDA)-approved drugs are palliative, aimed merely at relieving symptoms, and have been developed without recourse to knowledge of the underlying molecular mechanisms of disease [6]. This lack of target specificity is largely attributable to our lack of knowledge of the pathogenic mechanisms underlying most of these disease-associated genes and their mutations. There is thus an urgent need for systematic studies that provide insight into the mechanisms by which such mutations cause disease.

Previous studies have indicated that in-frame disease-associated coding mutations commonly alter protein-protein and protein-DNA interactions [7, 8], and that they preferentially perturb strong and stable biophysical interactions involved in key cellular processes [9]. Non-coding disease mutations have been reported to be enriched in DNase I hypersensitive sites and transcription factor binding motifs [10], and they have been shown to cause disease by disrupting transcriptional activation, *trans*-regulatory RNAs, splicing and translational regulation [11]. For instance, mutations in *cis*-regulatory elements have been found to exert a profound effect on carcinogenesis via differential transcription factor (TF) recruitment, altered binding kinetics or altered enhancer-promoter interactions [12]. Increasing evidence from chromatin conformation capture (3C)-based approaches, such as Hi-C [13], suggests that eukaryotic chromosomes are organized into higher order structures such as topologically associating domains (TADs) that are specified by DNA-binding proteins. These domains are vital for proper transcriptional regulation [14]; for example, their disruption has been implicated in oncogenic activation in gliomas [15]. Therefore, DNA-binding proteins including TFs can play multiple and complex roles in ensuring appropriate transcriptional regulation, and alterations of TF interactions through either coding or non-coding mutations can help to explain disease mechanisms [16]. Recently, pioneering high-throughput experiments have demonstrated how coding TF mutations affect TF-DNA interactions [17], further supporting the need for integrated analyses of both the protein and DNA components of transcriptional regulatory networks. Here, we construct the first three-dimensional integrated regulatory network (iRegNet3D) that combines TF-TF, TF-DNA and chromatin-chromatin interactions and TAD information to improve our understanding of the underlying pathogenic mechanisms of both coding and non-coding regulatory mutations. Based on the proteome-scale homology model approach we developed previously [7, 18, 19] and the Hi-C datasets, we have attempted to resolve the binding interfaces for all three types of interaction at high resolution in iRegNet3D. Furthermore, we have integrated the information of 50,877 coding and non-coding mutations into our database. We have built iRegNet3D as a web tool that allows users to query TFs or a list of mutations and see how the mutations affect network structure.

To study genetic mutations that alter gene regulation systematically, we compiled a list of disease-associated regulatory mutations from the Human Gene Mutation Database (HGMD) [20] including both missense coding mutations in TFs and non-coding mutations distributed throughout the genome. We find that disease-causing missense mutations in TFs are enriched both in protein-binding and DNA-binding interfaces, whereas non-coding disease-associated mutations are enriched at transcription start sites and enhancers. More generally, disease-associated mutations are found more frequently in TF binding motifs than are non-disease single nucleotide polymorphisms (SNPs) in the general population. Using Hi-C data, we show that non-coding mutation pairs across interacting chromatin regions are more likely to be associated with the same disease than mutation pairs across non-interacting regions. By integrating these interaction networks, we find that mutations in TF binding motifs across interacting loci of the same TF, or two motifs of interacting TFs, are more likely to cause the same disease. These results establish our iRegNet3D not only as a valuable resource to study the molecular mechanisms of both coding and non-coding regulatory mutations on a genomic scale, but also as an indispensable framework for interpreting the results of numerous ongoing large-scale sequencing and disease association studies.

## Results

### Construction of iRegNet3D

**Fig. 1:**
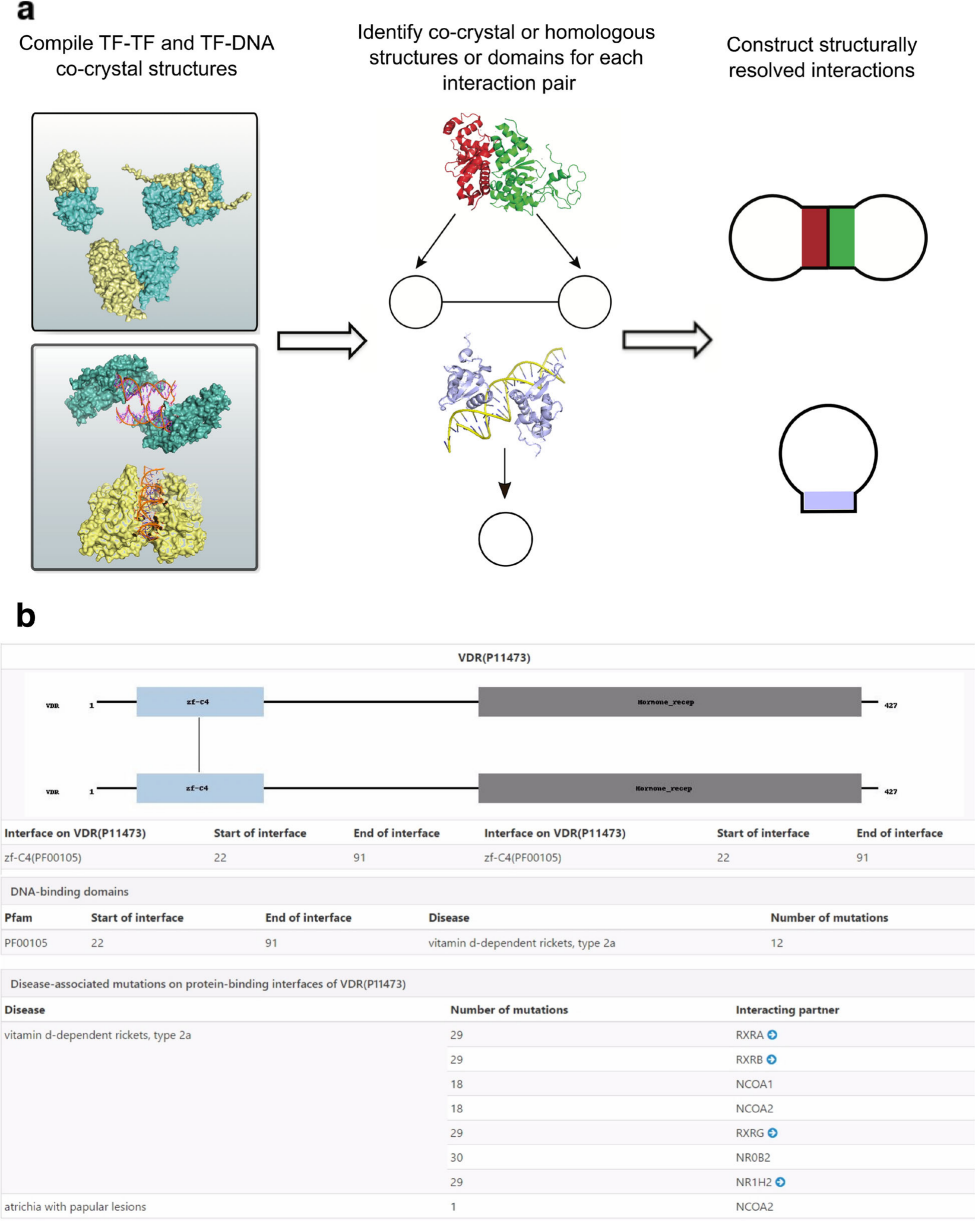
Construction and user interface of iRegNet3D. **a**. Homology modelling in the construction of iRegNet3D. **b** User interface of the iRegNet3D web tool showing the query page of the vitamin D receptor (VDR)

We previously compiled a list of experimentally validated high-quality binary protein-protein interactions in our HINT database [21]. We also collated a comprehensive list of experimentally validated and manually curated TFs from multiple sources [22–24]. To determine protein- and DNA-binding interfaces on these TFs, we used a homology modelling approach [7] for all amenable TF-TF and TF-DNA interactions in human, when co-crystal structures are not available (Fig. 1a). This resource-intensive process entails finding the most compatible co-crystal protein-protein and protein-DNA Protein Data Bank (PDB) structure (the template) for a given TF-TF or TF-DNA interaction (the targets) based on the sequence homology between the protein targets and all available PDB templates. We then prepared sequence alignments between the target protein sequences and the highest ranking template sequences. Interactions where either protein had coverage or sequence identity <40% were not considered amenable to modelling. We used MODELLER [25] to perform the actual homology modelling, which performs gap closing and insertions, and alleviates steric clashes through side-chain rearrangements. Finally, we evaluated models for the existence of knots, and eliminated any homology model that contained them. We also included available high-quality data on DNA-binding interfaces [22]. Overall, approximately 20% of the TF-TF and TF-DNA binding interfaces in iRegNet3D came from experimentally solved co-crystal structures, and 80% of the interfaces were inferred by our homology modelling method. For chromatin interactions, a list of intra-chromosomal chromatin interactions was obtained by combining anchor region information with target region information from the Hi-C data from [26]. We also integrated data of TADs from [26] into iRegNet3D. To facilitate the use of these data, we integrated the information of 50,877 coding and non-coding inherited disease-associated mutations [20] and built a web interface that allows users to query for specific disease-associated mutations as well as transcription factors. Users can visualize TF-TF interactions through modular diagrams, obtain the number of HGMD mutations located at each TF-TF and TF-DNA interface grouped by associated disease and traverse the TF-TF interaction network conveniently (Fig. 1b). Furthermore, users can upload a list of mutations, which our web tool will take as input and calculate a number of summary statistics including the number of coding mutations in TFs, the number of non-coding mutations, the fraction of mutation pairs across interacting TFs and the fraction of mutation pairs across interacting chromatin regions. Batch download is provided for our TF-TF interaction network, DNA-binding domain of TFs, chromatin interaction network and TAD boundaries. Our iRegNet3D web tool is now available at http://iregnet3d.yulab.org.

Our iRegNet3D is different from existing tools analysing regulatory networks. For example, iBIG [27] collected a number of regulatory networks including pathway interactions, protein-protein interactions and genetics interactions, and claimed to be a tool for building and visualizing regulatory networks especially from microarray data on human disease. However, it focuses more on building genome-wide networks perturbed by disease rather than identifying specific interactions disrupted by disease-associated mutations. Similar tools using gene regulatory networks include HumanNet [28] and MORPHIN [29]; however, these tools are aimed at discovering novel genes rather than explaining currently known disease mutations. Other existing tools focus on specific aspects of regulatory networks, such as protein-protein interactions as in the case of INstruct [19] and HINT [21] that we developed before, and gene-phenotype relationships as in the case of Phenolyzer [30]. To our knowledge, iRegNet3D is the only tool that integrates TF-TF interactions, TF-DNA interactions and chromatin-chromatin interactions as well as TADs to study mutation/gene-phenotype relationships and provide mechanistic insights of disease-associated mutations in both coding and non-coding regions.

### Disease-associated missense mutations in transcription factors are significantly enriched in interfaces that mediate protein or DNA binding

**Fig. 2:**
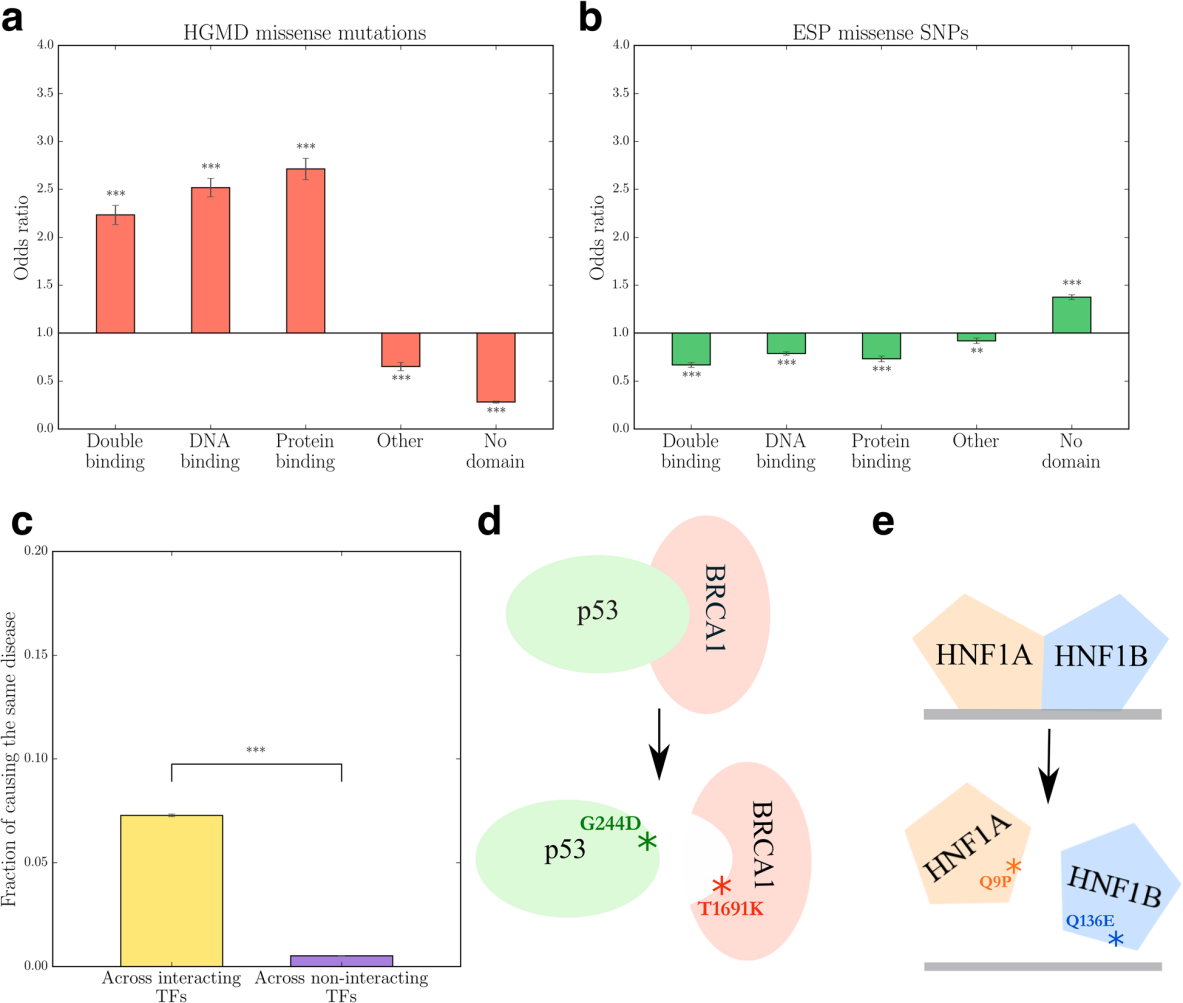
Analysis of disease-causing missense mutations on transcription factors. **a** Odds ratio for the distribution of transcription factor HGMD missense mutations in different interaction interfaces. ***P < 10^–3^. *P* values calculated using the Z-test on log odds ratio. Error bars indicate ± standard error (*SE*). **b** Odds ratio for the distribution of transcription factor ESP missense SNPs in different interaction interfaces. ***P < 10^–3^, **P < 10^–2^. *P* values calculated using the Z-test on log odds ratio. Error bars indicate ± SE. **c** Fraction of mutation pairs across two transcription factors causing the same disease. ***P < 10^–3^. Error bars indicate ± standard error of the mean (*SEM*). *P* values calculated using the cumulative binomial test. **d** Schematic diagram of a mutation pair causing the same disease across a TF-TF interaction interface. **e** Schematic diagram of a mutation pair causing the same disease where one mutation is on the TF-TF interaction interface while the other is on the TF-DNA interaction interface

The practical utility of iRegNet3D was first tested in investigating disease-associated missense mutations in transcription factors. Mutations within coding regions can be divided into in-frame mutations and frameshift mutations. The former category can be further partitioned into missense mutations and in-frame insertions or deletions. Missense mutations may cause disease by altering protein stability and aggregation [31], as well as by disrupting specific protein-protein interactions or protein-DNA interactions [32]. Coding mutations from the HGMD database are known to cause a variety of different diseases, most frequently affecting metabolism, development and the nervous system (Additional file 1). In iRegNet3D we resolved 7671 DNA-binding interfaces of all 1801 DNA-binding proteins, the majority of which are TFs, for both protein-protein and protein-DNA interactions at atomic resolution. Since many DNA-binding interfaces are known to simultaneously participate in protein-protein interactions, we considered these “double” interfaces that bind both DNA and protein as a separate category. We collected 3143 pathogenic missense mutations from HGMD and 17,507 missense SNPs from the National Heart, Lung, and Blood Institute (NHLBI) Exome Sequencing Project [33] that reside within the coding regions of transcription factors (TFs), and categorized them on the basis of the type of interaction interface in which they occur. We then calculated odds ratio values against the expected fraction of SNPs residing on each type of interface, which were derived as the fraction of amino acids belonging to that type of interface. The odds ratio measures the enrichment of disease mutations in a certain type of interface over random expectation. Although TFs are defined by their ability to bind DNA, we were surprised to discover that disease-associated mutations in TFs are more enriched on protein-protein interfaces than on protein-DNA interfaces (odds ratio = 2.23, *P* < 10^–3^ for 718 mutations on double binding interfaces; odds ratio = 2.71, *P* < 10^–3^ for 899 mutations on protein-binding interfaces; odds ratio = 2.52, *P* < 10^–3^ for 1140 mutations on DNA-binding interfaces; Fig. 2a), although all interaction interfaces exhibited significant enrichment. By contrast, SNPs from the general population that are not associated with deleterious effects are depleted in interfaces that mediate protein-protein or protein-DNA interactions (odds ratio = 0.67, *P* < 10^–3^ for 823 SNPs on double binding interfaces; odds ratio = 0.73, *P* < 10^–3^ for 697 SNPs on protein-binding interfaces; odds ratio = 0.79, *P* < 10^–3^ for 2222 SNPs on DNA-binding interfaces; Fig. 2b). These trends still hold even if only protein-protein and protein-DNA interactions with available co-crystal structures are employed (Additional file 2). Further, we found that mutation pairs across interacting TFs are much more likely (7.3% of pairs, *n* = 44,821) to cause the same disease than pairs across non-interacting TFs (0.52% of pairs, *n* = 8,670,342, *P* < 10^–3^; Fig. 2c). Taken together, these results suggest that the alteration of either protein-interacting or DNA-interacting interfaces on TFs is a common mechanism of disease. Identifying interface binding partners should help to establish novel disease genes and identify specific functions disrupted by the mutation leading to disease.

Interestingly, we find that two mutations across interacting TFs can cause the same disease in different ways. In our iRegNet3D analyses, we identified a total of 2036 mutation pairs on TF-TF interaction interfaces, and 3316 mutation pairs where one mutation is on the TF-TF interaction interface and the other is on the TF-DNA interaction interface. A pair of mutations located at the interaction interface between the two TFs could potentially disrupt or enhance their binding. For example, a missense mutation in *TP53* (c. 733G > A, G244D) and a missense mutation (c. 5191C > A, T1691K) in *BRCA1* have both been found to cause breast cancer [34, 35]. These two transcription factors are both involved in DNA damage repair, and they have been shown to interact with each other both physically and functionally [36, 37]. Both mutations are located at the interaction interface between these two proteins (Fig. 2d). The Gly 244 residue on p53 is located on its L3 loop, which has been shown to interact with the Brca1 C-terminal (BRCT) domain of BRCA1. Thr 1691 of BRCA1 is located at the BRCT domain between β3 and α2 [35]. Therefore, alterations to the binding between these two proteins might constitute the mechanism by which these mutations cause breast cancer.

An alternative mechanism can be demonstrated by a missense mutation in HNF1A (c.26A > C, Q9P) and a missense mutation in HNF1B (c.406C > G, Q136E). Both mutations cause maturity-onset diabetes of the young (MODY) [38, 39], and HNF1A forms heterodimers with HNF1B through their N-terminal dimerization interfaces [40–42]. The former mutation is located at the dimerization interface of HNF1A and may lead to the abolition of its heterodimerization with HNF1B, whereas the latter mutation is located at the DNA-binding interface of HNF1B (Fig. 2e). The Q136E mutant protein has been shown to have no detectable DNA-binding ability [43]. Because dimerization is required for members of the HNF1 homeoprotein family of transcription factors to bind DNA [44, 45], the alteration of the heterodimerization between HNF1A and HNF1B and the abolition of the DNA-binding activity of HNF1B have essentially the same impact on transcriptional regulation; hence both lead to MODY.

These findings have served to identify potential mechanisms by which two different and non-allelic mutations can cause the same disease. They also highlight the importance of integrating different types of molecular interactions as a means to fully understand the mechanisms of pathogenic regulatory mutations. Careful examination of such mutations within the framework of iRegNet3D may shed new light on these mutations and the means by which they give rise to the corresponding disorders at the molecular level. These mechanistic models will provide critical insights to design follow-up studies and experimental validations.

### Non-coding regulatory mutations across interacting chromosomal regions tend to be associated with the same disease

**Fig. 3:**
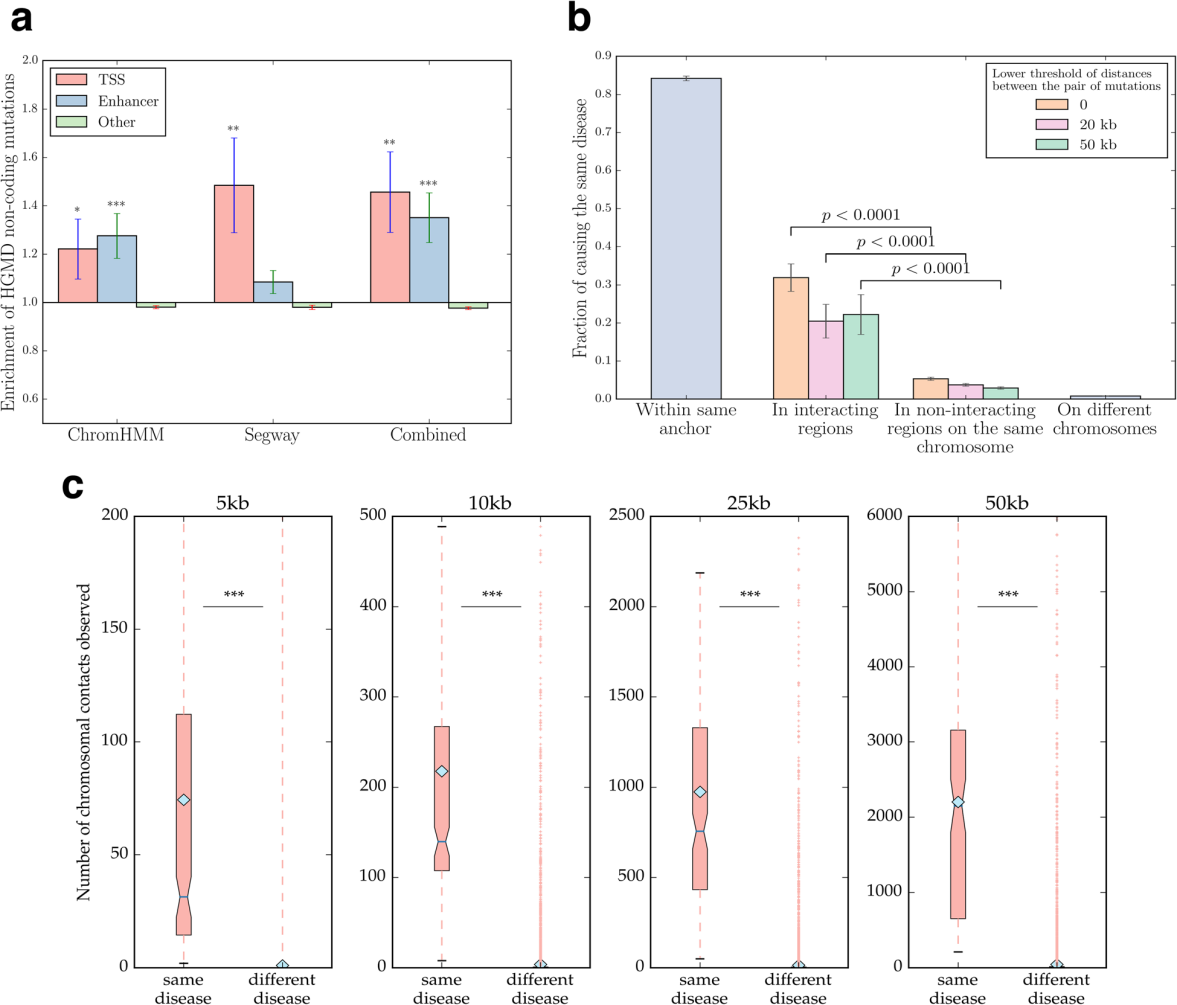
Analysis of disease-associated non-coding mutations and their locus heterogeneity. **a** Enrichment of HGMD non-coding mutations in different gene regulatory regions. *P < 0.05, **P < 10^–2^, ***P < 10^–3^. *P* values calculated using the Z-test on log enrichment. Error bars indicate ± SE. **b** Fraction of HGMD non-coding mutation pairs causing the same disease. Error bars indicate ± SEM. *P* values calculated using the cumulative binomial test. **c** Normalized number of chromosomal contacts for mutation pairs associated with the same disease or different diseases. ***P < 10^–3^. *P* values calculated using the Mann-Whitney U test. *Light blue dots* indicate the mean. *Dark blue lines* indicate the median

A large number of non-coding mutations have been identified and implicated in the pathogenesis of a variety of different diseases, including cancer [46]. Genome-wide association studies (GWASs) and quantitative trait loci (QTL) studies have been widely applied to the identification and annotation of non-coding mutations [47]. Previous studies have found that Mendelian disease mutations and recurrent cancer somatic mutations are enriched within promoter regions [48]. Enhancers in the human genome are also prone to deactivating mutations that disrupt the binding of transcription factors [49]. To study the localization of non-coding disease-associated mutations across the human genome, we categorized 2594 HGMD non-coding mutations using chromatin state annotation data from ENCODE. These mutations are most frequently associated with cancer, developmental disease and diseases of the digestive system (Additional file 1). We observed that mutations in non-coding regions (Additional file 3) are significantly enriched in transcription start sites and enhancers (Fig. 3a), consistent with their presumed role in transcriptional regulation.

Since increasing evidence has emerged for distal enhancers coming into close proximity with promoters via a looping mechanism [50–53] and their interaction is fundamental to the control of transcriptional activity [54], we sought to explore how chromatin interactions might be related to the phenotypic impact of non-coding mutations using iRegNet3D. We classified each pair of non-coding mutations as ‘in the same anchor’ (*n* = 3480), ‘across interacting regions’ (*n* = 166), ‘across non-interacting regions of the same chromosome’ (*n* = 3164) or ‘on different chromosomes’ (*n* = 1,128,161) using published 3D chromatin interactome data [26]. Consistent with the idea that mutations in the same anchor region are likely to affect the same regulatory element, we find these pairs to have an 80% probability of being associated with the same disease (Fig. 3b). Notably, two mutations across interacting chromatin regions have a significantly higher chance of causing the same disease than two mutations in non-interacting regions of the same chromosome (*P* < 10^–40^, cumulative binomial test; Fig. 3b). To eliminate the confounding influence of proximal interacting regions that could in fact be part of a single regulatory element, we required a minimum distance between the two mutations. We were able to obtain the same results when we selected a threshold of 20 kb (*P* < 10^–13^, cumulative binomial test; Fig. 3b) or even 50 kb (*P* < 10^–16^, cumulative binomial test; Fig. 3b).

To further validate our results, we used a more recent 3D map of the human genome constructed using Hi-C [55], and investigated whether mutations that cause the same disease tend to be located in regions that have a high contact frequency. Indeed, higher contact numbers were observed between regions across which mutations cause the same disease as compared to regions across which mutations cause different diseases, irrespective of the resolution of the data used (Fig. 3c). These results strongly suggest that many non-coding disease mutations can affect the interactions between distal and proximal regulatory elements. Indeed, instances have been reported where single nucleotide variants either disrupt or strengthen promoter-enhancer interactions, thereby altering the transcriptional activity of the regulated gene [56]. As an example, the single nucleotide polymorphism (SNP) rs12913832 is located within a postulated enhancer of *OCA2*. It has been shown using 3C that there is a stronger interaction between the enhancer and the *OCA2* promoter for the T pigmentation-associated allele than that observed for the pigmentation-non-associated C allele [56].

Importantly, Hi-C studies have found that many chromatin-chromatin interactions and topologically associating domains are cell-type independent [57], thereby rendering cell type matching unnecessary for many such interactions. Nevertheless, as Hi-C data become available from more cell types, our approach would benefit from using only mutations associated with diseases that are matched for the cell type used for Hi-C analysis.

### Analysis of non-coding disease-associated mutations that alter TF binding motifs indicates alterations of chromatin looping

**Fig. 4:**
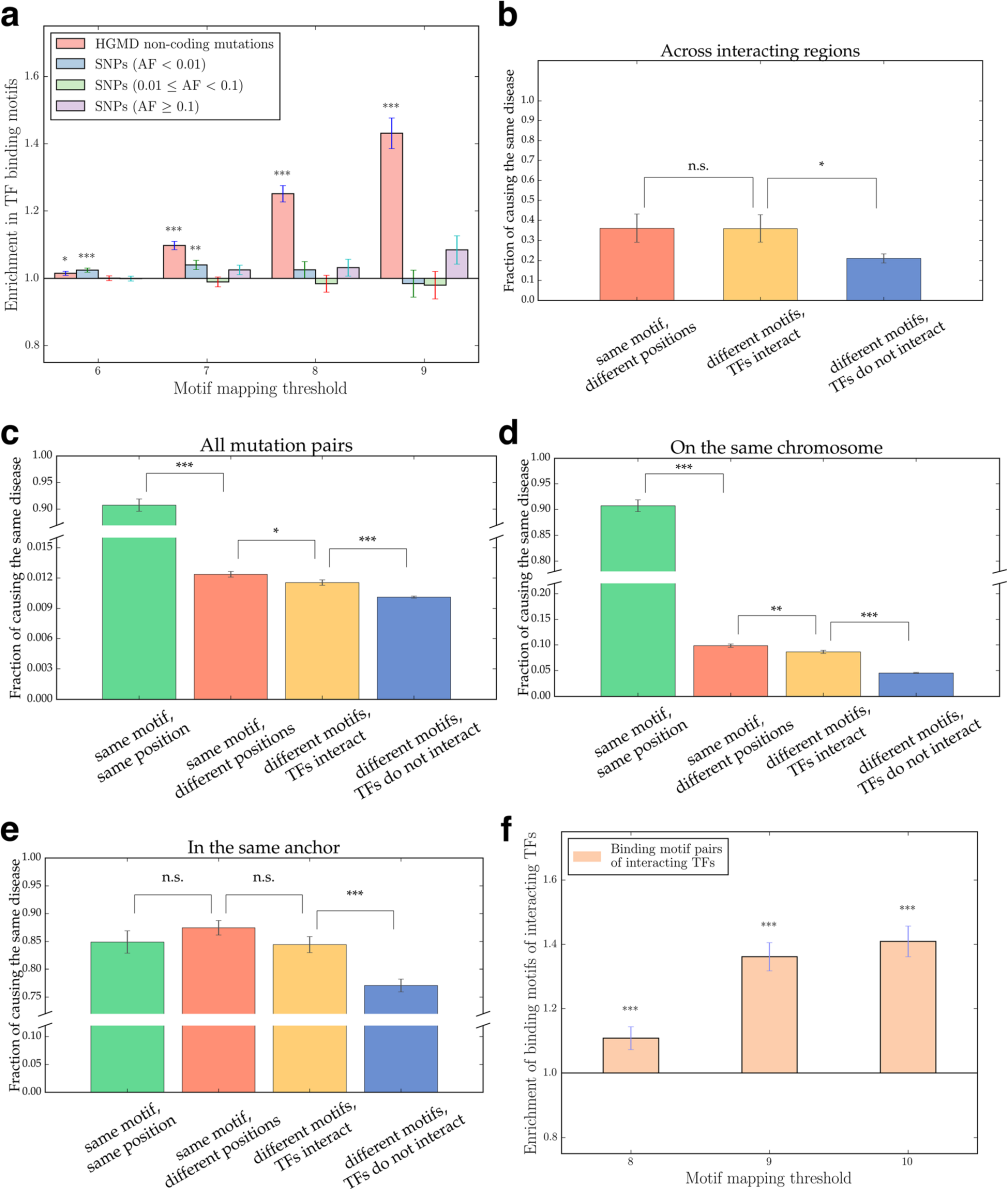
Analysis of disease-associated non-coding mutations located at TF binding motifs. **a** Enrichment of HGMD non-coding mutations and population SNPs in TF binding motifs. *P < 0.05, **P < 10^–2^, ***P < 10^–3^. *P* values calculated using the Z-test on log enrichment. Error bars indicate ± SE. **b** Fraction of TF binding motif-localized non-coding mutation pairs causing the same disease. Error bars indicate ± SEM. *P < 0.05. *P* values calculated using the cumulative binomial test. *n.s.* not significant. **c** Fraction of TF binding motif-localized non-coding mutation pairs on the same chromosome causing the same disease. Error bars indicate ± SEM. *P < 0.05, ***P < 10^–3^. *P* values calculated using the cumulative binomial test. **d** Fraction of TF binding motif-localized non-coding mutation pairs in the same anchor causing the same disease. Error bars indicate ± SEM. **P < 10^–2^, ***P < 10^–3^. *n.s.* not significant. *P* values calculated using the cumulative binomial test. **e** Fraction of TF binding motif-localized non-coding mutation pairs across interacting regions causing the same disease. Error bars indicate ± SEM. ***P < 10^–3^. *n.s.* not significant. *P* values calculated using the cumulative binomial test. **f** Enrichment of motif pairs of interacting TFs across interacting chromatin regions. Error bars indicate ± SEM. ***P < 10^–3^. *P* values calculated using the Z-test on log enrichment

Many types of transcriptional regulation are mediated by transcription factors (TFs). To determine whether there is a tendency for non-coding mutations to alter TF binding to regulatory elements, we scanned for known TF binding motifs in genomic regions where the mutations are located using the RTFBSDB package [58]. Perhaps unsurprisingly, non-coding disease mutations were found to be enriched in TF binding motifs, whereas population SNPs were not (Fig. 4a). In addition, as the log-likelihood cutoff score used for identifying TF motifs increases, the fold of enrichment becomes higher (Fig. 4a). This is suggestive of an important role for alterations of TF binding sites in the pathogenesis of human genetic disorders. We have performed the same calculation again using only the subset of mutations located across interacting chromatin regions, and found that these mutations are also enriched in TF binding sites (*P* = 0.017).

In addition to affecting TF-DNA binding, non-coding mutations may also disrupt or enhance those chromatin interactions that are mediated by TFs. Usually, chromatin looping is facilitated by a TF multimer that binds to distal motifs. For example, it is known that loops mediated by CTCF dimers play a complex regulatory role in transcription regulation [59, 60]. Additionally, multiple TFs can form or recruit a complex to create chromatin loops. For example, TF recruitment of RNA polymerase II may mediate chromatin looping [61].

Since non-coding mutations may affect these TF-mediated chromatin interactions, we focused on mutations in TF binding motifs at interacting chromosomal loci. We classified each mutation pair based on the distance and interactions between their corresponding TF motifs. Two mutations could be located within the same TF binding motif (‘same motif, same position’), at binding motifs of the same TF at different locations (‘same motif, different position’), at binding motifs of two different TFs that interact physically (‘different motifs, TFs interact’) or at binding motifs of two non-interacting TFs (‘different motifs, TFs do not interact’). Within each group, we calculated the fraction of mutation pairs causing the same disorder. Unsurprisingly, two mutations in a single TF binding motif have a >80% possibility of causing the same disorder (Fig. 4c–e). In addition, mutation pairs across interacting DNA regions located at different binding sites of the same TF, as well as those located at binding sites of interacting TFs, are all significantly more likely to be associated with the same disorder than mutation pairs across interacting regions located at binding sites of non-interacting TFs (Fig. 4b). This result was found to be robust irrespective of whether the analysis was performed for all mutation pairs (Fig. 4c), mutation pairs on the same chromosome (Fig. 4d) or only mutation pairs in the same anchor (Fig. 4e) that already have a high baseline probability of causing the same disease. To determine if TF-TF interactions play an important role in mediating chromatin-chromatin interactions, we took 4-kb windows centred at mutations in interacting chromatin regions, and scanned for TF binding motifs. Among all motif pairs across interacting chromatin regions, we discovered that there is an enrichment of motifs of interacting TFs, and that this enrichment increases as the matching of TF binding motifs becomes more stringent (Fig. 4f). We further validated this result using an alternative null model based on the fraction of interacting TF motif pairs from a scrambled chromatin interaction network. These results indicate that TF-mediated chromatin looping may be important for understanding disease mechanisms, and meaningful TF-TF interactions may be encoded in the DNA sequences of regions involved in making chromosomal contacts.

**Fig. 5:**
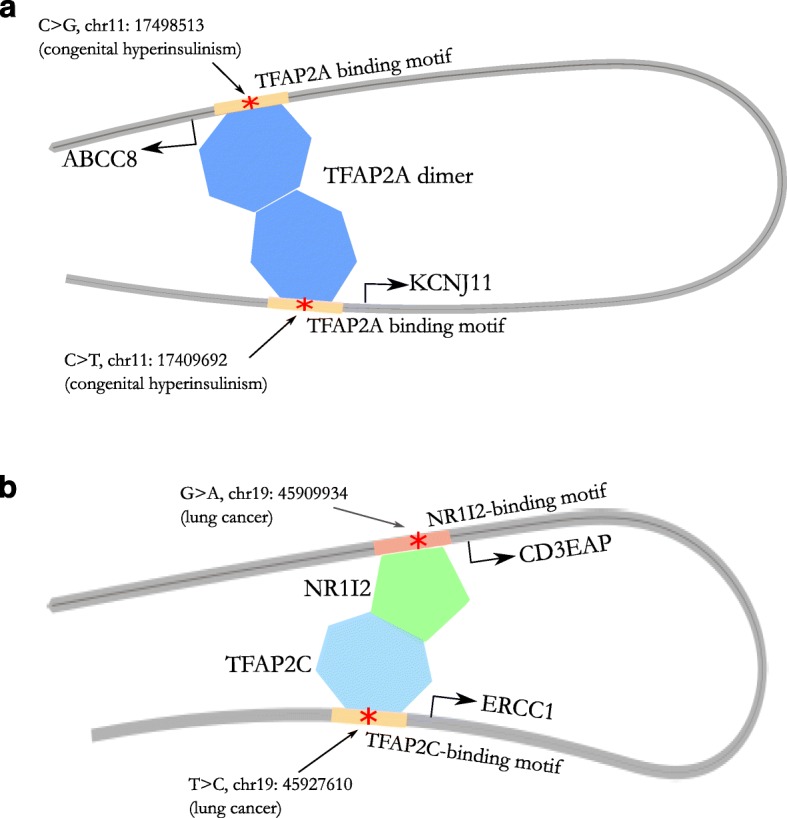
Mutations across interacting chromatin regions cause diseases by potentially disrupting TF-mediated chromatin looping. **a** Schematic diagram of two mutations across interacting TF regions located at the same type of TF binding motif, and causing the same disease. **b** Schematic diagram of two mutations across interacting TF regions located at two binding motifs of TFs that interact with each other, causing the same disease

An interesting example of two mutations across interacting DNA regions located within two distinct binding sites for the same TF (Fig. 5a) is to be found on chromosome 11. Both of the mutations in question give rise to congenital hyperinsulinism, characterized by dysregulated insulin secretion and hypoglycemia, and they were reported in two separate clinical studies [62, 63]. One of the mutations (C to G; chr11: 17498513, hg19) is located in the promoter region of the *ABCC8* gene, 64 base pairs upstream of the transcriptional initiation site. The other (C to T; chr11: 17409692, hg19) is located 54 base pairs upstream of the start codon of *KCNJ11*. The two mutations are around 90 kb apart. Both mutations are located within putative TFAP2A-binding motifs, and the two chromatin loci interact. ABCC8 and KCNJ11 contribute subunits to the β-cell ATP-sensitive K^+^ channel (K_ATP_), whose activity is dependent upon the ATP/ADP ratio and serves to regulate insulin secretion [63]. A number of coding mutations as well as intronic mutations in the two genes have also been reported to cause congenital hyperinsulinism [64]. In addition, TFAP2A belongs to the AP-2 family of transcription factors that has been reported to bind to estrogen receptor alpha to facilitate long-range chromatin interaction and transcription [65]. Further, a previous study has shown that TFAP2A overexpression can lead to increased insulin receptor expression [66], possibly disrupting insulin metabolism. It is therefore likely that *ABCC8* and *KCNJ11* are co-regulated as a result of TFAP2A-mediated chromatin looping, and that the disruption of TFAP2A binding at either locus leads to congenital hyperinsulinism.

Mutations across interacting DNA regions causing the same disease have also been found to be located within binding sites of interacting TFs (Fig. 5b). Two mutations on chromosome 19 have been reported to be associated with susceptibility to lung cancer. One of them (T to C; chr19: 45927610, hg19) is located in the promoter of the *ERCC1* gene about 1 kb upstream of the start codon, and has been reported to affect transcriptional regulation of ERCC1 [67]. The other (G to A; chr19: 45909934, hg19) is located 21 base pairs upstream of the start codon of the *CD3EAP* gene, and the mutant allele has increased promoter activity and is associated with increased expression of CD3EAP [68]. The former mutation is within a putative TFAP2C-binding site, whereas the latter is located within a putative NR1I2-binding site. The two chromatin loci (~18 kb away) interact according to Hi-C data, whilst the protein-protein interaction between TFAP2C and NR1I2 has been reported previously [69]. As the limiting factor in nucleotide excision repair, the expression level of ERCC1 has been shown to be associated with survival outcome in non-small-cell lung cancer [70]. Interestingly, reduced expression of NR1I2 has also been shown to increase the risk of lung cancer [71], consistent with an important role for this TF in the normal expression of ERCC1. On the other side, enhanced TFAP2C expression has been shown to promote lung tumorigenesis and aggressiveness [72]. Considering the fact that AP-2 family TFs mediate chromatin looping [65], it may be that the NR1I2-TFAP2C complex facilitates chromatin looping at this locus in order to regulate ERCC1 and CD3EAP, and the disruption of this regulation contributes to lung tumorigenesis. It is highly interesting to perform further experiments, such as ChIP-seq studies of TFAP2A, TFAP2C and NR1I2 in relevant cell types, to validate these hypotheses generated by our iRegNet3D models.

## Discussion

Although, with the advent of high-throughput sequencing, many disease-associated mutations have been identified, there have been very few analyses that capture both coding and non-coding mutations in a single genome-wide framework. Here, we constructed an integrated regulatory network, iRegNet3D, that encompasses TF-TF, TF-DNA and chromatin interactions as well as topologically associating domains (TADs). iRegNet3D provides a user-friendly web interface that allows users to query TFs and disease-associated mutations to examine how the regulatory network structure is perturbed. Using a high-quality list of disease mutations, we have traced pathogenic mechanisms to the interface of protein- and DNA-interaction networks. Specifically, we find significant enrichment of missense mutations in both protein-binding and DNA-binding interfaces of TFs, as well as in the TF binding sites at transcription start sites and enhancers. Similarly, mutations across the same interface of a chromatin loop are more likely to be associated with the same phenotypic effect than mutations in non-interacting chromatin regions. In line with previous findings, our data reinforce chromatin looping as an informative regulatory paradigm that is likely to be disrupted by many pathogenic non-coding mutations.

Importantly, the models we proposed in cases where mutation pairs are located at binding motifs of interacting TFs are not the only possibilities. One alternative scenario is that nearby factors facilitate chromatin looping, instead of the specific TF binding sites we propose here. This would instead suggest that the disease mutation pairs cause similar defects in transactivation but not chromatin contacts. Hi-C data from mutant cell lines would be required to discern if the mutations have a disruptive effect on chromatin interactions. RNA-seq or qPCR of nearby target genes would serve to confirm aberrant transcriptional regulation, whilst ChIP-seq data would confirm aberrant factor binding to motifs that may be disrupted by the mutations.

Our current analyses are primarily limited by the available number of high-quality coding and non-coding mutations for which we have direct clinical or functional evidence for their association with specific human disorders. As sequencing continues to become cheaper, additional disease mutations can be incorporated into our analysis framework and should help to generate new insights into the transcription regulatory network architecture. A more comprehensive protein-protein, protein-DNA and DNA-DNA interaction network would also increase the coverage and depth of our study.

Intriguingly, we have observed that many non-interacting TF pairs that cause the same disease are linked in another way: one TF binds the enhancer or promoter of the other. We have attempted to perform a systematic analysis to explore whether missense mutations in TFs and non-coding mutations at their binding sites tend to cause the same disease. Unfortunately, there are currently insufficient mutation data to draw any statistically meaningful conclusions. However, with the rapidly increasing number of disease mutations and chromatin interaction maps being reported, we plan to perform these analyses in the near future.

Overall, our iRegNet3D framework provides new insights into the mechanisms by which coding and non-coding regulatory mutations disrupt network structure and cause various diseases at the molecular level. This is of great importance for the design of experimental follow-up studies to further our understanding of these disease genes and their mutations. With the rapidly developing genome editing technologies such as CRISPR [73, 74], various molecular and functional experiments can be designed to validate the disease-causing mechanisms of coding and non-coding regulatory mutations that are predicted by iRegNet3D. Furthermore, iRegNet3D promises to be an indispensable tool for numerous ongoing large-scale sequencing projects and genome-wide association studies (GWASs) to link poorly understood disease genes and mutations. Although GWASs have been hugely successful in identifying variant-trait associations, they are often underpowered to pinpoint the exact causal variant. iRegNet3D can be used to generate mechanistic hypotheses by presenting all known connections to other chromatin regions, TFs, and mutations that cause the same disease. It can be used in conjunction with existing functional scoring tools such as Combined Annotation-Dependent Depletion (CADD) [75], Eigen [76] and FunSeq2 [77] to identify variants that lead to the phenotype. Specifically, starting from a list of variants found by GWASs, one can pick out the potentially causal ones by applying a filter of the functional scores, and use iRegNet3D to generate possible models of disruption that could be tested at the molecular level. Conversely, if medical genomics outpaces interactome discovery, disease mutation pairs could be mined to predict TF-TF, TF-DNA or chromatin-chromatin interactions. The mechanistic insights provided by our iRegNet3D framework have the potential to greatly increase the explanatory power of association studies, thereby helping us to achieve better accuracy and coverage in disease mutation discovery.

## Conclusions

Here we present iRegNet3D, an integrated regulatory network incorporating TF-TF, TF-DNA, chromatin interactions and TAD information at high resolution. To our knowledge, it is the only tool that integrates multiple regulatory networks and human disease-associated mutations for the generation of mechanistic insights into pathogenesis. Using iRegNet3D, we have demonstrated that disease-causing missense mutations on TFs are enriched in protein-binding and DNA-binding interfaces. On the other hand, disease-associated non-coding mutations tend to impact promoters and enhancers, and many of them alter TF binding motifs. Most importantly, we have found that disruption of chromatin looping through TF-TF interactions is potentially a mechanism by which mutation pairs can cause the same disease, and that either homo- or heterodimeric TF-TF interactions could be involved. iRegNet3D provides a framework and a user-friendly web tool for understanding the mechanisms by which both coding and non-coding mutations lead to disease, and may facilitate the future discovery of hitherto unknown disease genes and mutations.

## Methods

### Homology modelling of TF-TF interaction and DNA-binding interfaces of TFs

Potential co-crystal templates for homology modelling were ranked by the coverage and sequence identity of the target proteins to the template. Only interactions where either protein has coverage or sequence identity above 40% were considered amenable to modelling. Interactions with a single viable template were modelled using that template, and models with more than one template were modelled with the single template with the highest match score. The match score takes into account both sequence identity and coverage of each target protein to the template: m = SeqID1*Cov1 + SeqID2*Cov2. MODELLER [25] was used for actual modelling. Any protein domains that contained interaction residues as predicted by the models were considered interaction interfaces.

To verify the reliability of our inferred TF-TF and TF-DNA interfaces, we performed a threefold cross-validation, similar to what was described in [7], on the sets of TF-TF and TF-DNA interactions separately for which we have co-crystal structures. We split the interactions into three subsets where co-crystal structures in the first two subsets were used as templates to infer TF-TF or TF-DNA interfaces in the third subset. We repeated the procedure three times for all three training-testing divisions. More than 90% of all the TF-TF or TF-DNA interfaces could be correctly predicted with our method.

### Enrichment of TF missense mutations on protein-binding and DNA-binding interfaces

Only TFs containing at least one protein-protein and one protein-DNA interface were included in order to minimize misclassifications due to incomplete TF annotations. Inherited disease-causing coding mutations were obtained from HGMD’s curated ’DM’ category. Missense SNPs were taken from the Exome Sequencing Project if their allele frequency was greater than 1%. We reproduced our missense SNP results at multiple allele frequency thresholds or using data from the 1000 Genomes Project (data not shown). Protein interaction interfaces were collected from our 3D protein interaction network (hSIN); we also validated our results using only protein interaction interfaces with available crystal structures (hSIN co-crystal set; Additional file 2). For each type of interaction interface, the total numbers of variants and amino acids were counted. Finally, the fractions of amino acids and mutations were computed (compared to all mutations or all amino acids, respectively) and used to calculate odds ratios. The formula describing the odds ratio is as follows:

$$ \mathrm{OR}=\frac{{\mathrm{p}}_1/\left(1-{\mathrm{p}}_1\right)}{{\mathrm{p}}_2/\left(1-{\mathrm{p}}_2\right)}, $$

where *p*_1_ is the fraction of mutations located at a type of interface (*n*_mut, region_/*n*_mut, total_), and *p*_2_ is the fraction of amino acid residues that belong to that type of interface (*n*_res, region_/*n*_res, total_). Z scores for odds ratios were calculated as follows:

$$ S{E}_{lnOR} = \sqrt{\frac{1}{n_{mut,\  region}} + \frac{1}{n_{mut,\  other}} + \frac{1}{n_{res,\  region}} + \frac{1}{n_{res,\  other}}} $$

$$ Z = \frac{ \ln (OR)}{S{E}_{\ln OR}} $$

### Enrichment of non-coding mutations on promoters and enhancers

Non-coding disease-associated mutations were obtained from HGMD (accessed February 2015); only mutations within the DM, DM?, DFP and DP categories were used for analyses. Chromatin segregation data using ChromHMM, Segway and a combined method were obtained from ENCODE [78] for several cell lines. For ChromHMM annotations, Tss and TssF were regarded as TSS segments and Enh, EnhF were regarded as enhancers. For Segway annotations, Tss and TssF were regarded as TSS segments and Enh, Enh1, Enh2, EnhF, EnhF1, EnhF2, EnhF3, EnhP and EnhPr were regarded as enhancers. For combined annotations, TSS was regarded as TSS segments and E was regarded as enhancers. TSS regions in different cell lines were combined, and enhancers in different cell lines were combined. Enrichment was calculated as (*n*_mut, region_ * *l*_total_)/(*n*_mut, total_ * *l*_region_), where *n*_mut, region_ is the number of mutations in that type of chromatin segment, *n*_mut, total_ is the total number of mutations, *l*_region_ is the total length of that type of segment in all the chromosomes and *l*_total_ is the total length of all chromosomes. Z scores for enrichment values were calculated as follows:

$$ S{E}_{ln\  Enrichment} = \sqrt{\frac{1}{n_{mut,\  region}}\mathit{\hbox{-}}\frac{1}{n_{mut,\  total}}+\frac{1}{l_{region}}\mathit{\hbox{-}}\frac{1}{l_{total}}\ } $$

$$ Z=\frac{ln(Enrichment)}{S{E}_{logEnrichment}} $$

### Statistical analysis of mutation pairs and their phenotypes: coding mutations

We used the previously constructed high-quality interactome INstruct [19] to determine if two proteins interact. For mutation pairs across two different TFs, we determined if a mutation pair was ‘across interacting TFs’ or ‘across non-interacting TFs’ by checking whether the two TFs at which the mutations are localized interact with each other in hSIN. We then calculated the fraction of mutation pairs causing the same disease in these two categories. The statistical significance between the two categories was calculated using the cumulative binomial test.

### TF binding motif mapping

Common SNPs were obtained from the UCSC Genome Annotation database and partitioned by allele frequency. From each category, 2000 SNPs were randomly selected. The RTFBSDB R package [58] was used to search for TF binding motifs within which the HGMD non-coding mutations and common SNPs were located. Log-likelihood score thresholds of 6, 7, 8 and 9 were used to identify TF binding motifs.

### Enrichment of non-coding mutations in TF binding motifs

Regions of 4000 bp (search regions) centred at non-coding mutations, as well as population SNPs selected, were used for TF binding motif scanning. For a given mapping threshold (6, 7, 8 or 9), enrichment of non-coding mutations and population SNPs were calculated as (*n*_var, motif_ * *l*_motifs in search_ _region_) / (*n*_var, total_ * *l*_search region_), where *n*_var, motif_ is the number of mutations or variants in at least one TF binding motif, *n*_var, total_ is the total number of mutations or variants, *l*_motifs in search_ _region_ is the total length of TF binding motifs within search regions for that type of mutation or variant and *l*_search region_ is the total length of all the search regions for that type of mutation or variant. Standard errors and Z scores were calculated similarly to the calculation above. We performed this for all four types of mutation or variant: HGMD non-coding mutations, population SNPs with minor allele frequencies less than 0.01, population SNPs with minor allele frequencies between 0.01 and 0.1 and population SNPs with minor allele frequencies greater than 0.1.

### Statistical analysis of mutation pairs and their phenotypes: non-coding mutations

Disease names were mapped to Medical Subject Headings (MeSH) Unique IDs or Online Mendelian Inheritance in Man (OMIM) IDs using DNorm [79] (version 0.0.6). Only mutations whose associated disease names were successfully mapped were retained; the final list contained 1666 mutations. Chromatin interaction data that contained a list of ‘anchors’, and a list of interactions between chromatin regions and anchors, were obtained from [26]. All the interactions in these files were intra-chromosomal. Each pair of non-coding mutations was classified as ‘in the same anchor’ if both mutations were located at the same anchor in the anchor list, ‘in interacting regions’ if one of the mutations was in an anchor and the other was in a region interacting with that anchor, ‘in non-interacting regions on the same chromosome’ if it did not belong to the previous two categories but was located on the same chromosome and ‘on different chromosomes’ if the two mutations were located on two different chromosomes. For the ‘in interacting regions’ and ‘in non-interacting regions on the same chromosome’ categories, we required that the distance between the two mutations must be smaller than 2 Mb, and greater than 0 kb, 2 kb or 5 kb for three different analyses. The fractions of mutation pairs associated with the same disease were calculated, and *P* values were calculated using the cumulative binomial test.

For the comparison of chromatin interactions (Fig. 3c), we obtained high-resolution Hi-C data from [55]. We used the intra-chromosomal interaction data with resolutions of 5 kb, 10 kb, 25 kb and 50 kb. We labelled mutation pairs on the same chromosome as being associated with the same disease or different diseases. For each mutation pair, we located the corresponding chromosomal regions and calculated the SQRTVC normalized number of chromatin contacts by means of the raw matrix and SQRTVC normalization vector provided in the data. Statistical significance between the two groups was then calculated using the Mann-Whitney U test.

For the analysis of non-coding mutations at TF binding sites, we used the TF binding motif scanning result with a default threshold of 6. We performed calculations for all mutation pairs, mutation pairs on the same chromosome, mutation pairs in the same anchor (as defined by the chromatin interaction data used above) and mutation pairs across interacting regions. Mutation pairs across interacting regions were defined using Hi-C data from [26, 80], GSM2101551, EMBL-EBI sample E-GEOD-77266 and 5-C data from [52]. All of these datasets contain lists of interacting chromatin regions. A mutation pair was termed ‘interacting’ if one of the mutations was located in a region in the list and the other was localized to a region on the same chromosome that interacts with the previous region. For each calculation, only mutation pairs where both mutations were located in at least one TF binding motif were retained. Mutation pairs were labelled ‘same motif, same position’ if the two mutations were located within exactly the same TF binding site, ‘same motif, different positions’ if the two mutations were located within the binding sites of the same TF but at different chromosomal positions, ‘different motifs, TFs interact’ if the two mutations were located at the binding motifs of two TFs that interact with each other as determined by INstruct and ‘different motifs, TFs do not interact’ if the two mutations were located at binding motifs of two TFs that do not interact with each other. Fractions of mutation pairs causing the same disease were calculated, and statistical significance was calculated using the cumulative binomial test.

### Enrichment of binding motifs of interacting TFs across interacting chromatin regions

Regions of 4000 bp centred at HGMD mutations selected from mutation pairs across interacting regions were used for TF binding motif search. The RTFBSDB R package was used, and we employed cutoff scores of 8, 9 and 10 due to the large number of TF binding motifs found at lower thresholds. For each pair of TF binding motifs across interacting chromatin regions, we determined whether the corresponding TFs interact with each other using the TF-TF interaction network of iRegNet3D. The expected fraction of motif pairs whose corresponding TFs interact was calculated by dividing the number of interacting TF pairs by the number of all possible TF pairs where both of the TFs are involved in at least one TF-TF interaction. Enrichment of motifs of interacting TFs was calculated against this baseline, and *P* values were calculated using the Z-test on log enrichment value as described above. The alternative null model was built by scrambling the chromatin interaction network to produce motif pairs across non-interacting regions. Enrichment of motif pairs of interacting TFs across interacting chromatin regions was calculated against the fraction of motif pairs of interacting TFs in this null model.

## Additional files

Additional file 1: Supplementary figure: a. Disease group annotation of HGMD coding missense mutations. b. Disease group annotation of HGMD non-coding regulatory mutations. (PDF 1311 kb)
Additional file 2: Supplementary figure: a. Odds ratio for the distribution of transcription factor HGMD missense mutations in different interaction interfaces using only interfaces with co-crystal structures. ****P* < 10^–3^. *P* values calculated using the Z-test on log odds ratio. Error bars indicate ± standard error (SE). b. Odds ratio for the distribution of transcription factor ESP missense SNPs in different interaction interfaces using only interfaces with co-crystal structures. Error bars indicate ± SE. (PDF 301 kb)
Additional file 3: Supplementary tables: summary statistics of iRegNet3D. (XLSX 29 kb)
