# Supplementary figures and images for: iRegNet3D: three-dimensional integrated regulatory network for the genomic analysis of coding and non-coding disease mutations
Source: Genome Biol. 2017 Jan 18;18:10. doi: 10.1186/s13059-016-1138-2 (PMC5241969; doi:10.1186/s13059-016-1138-2)

**a**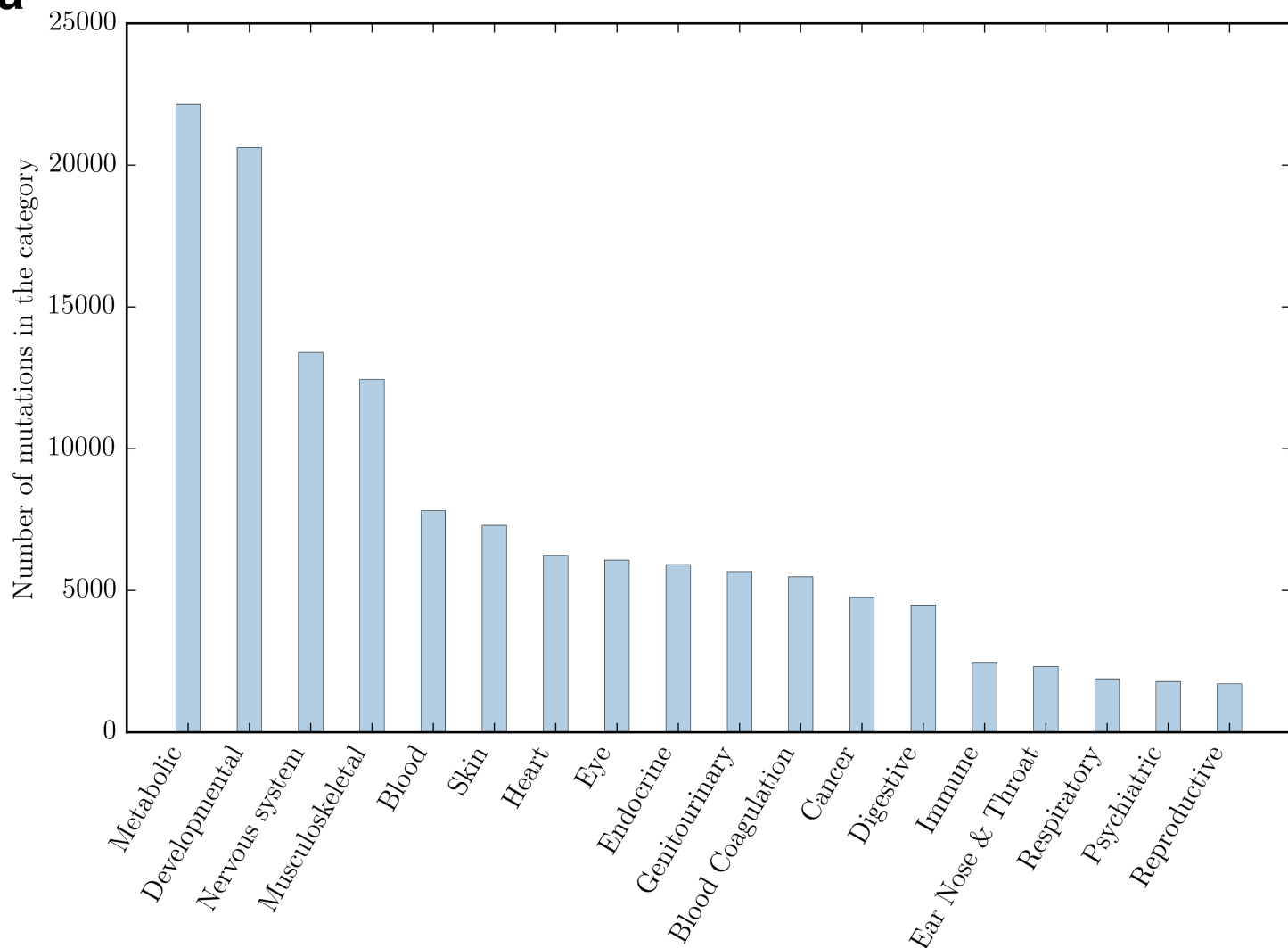**b**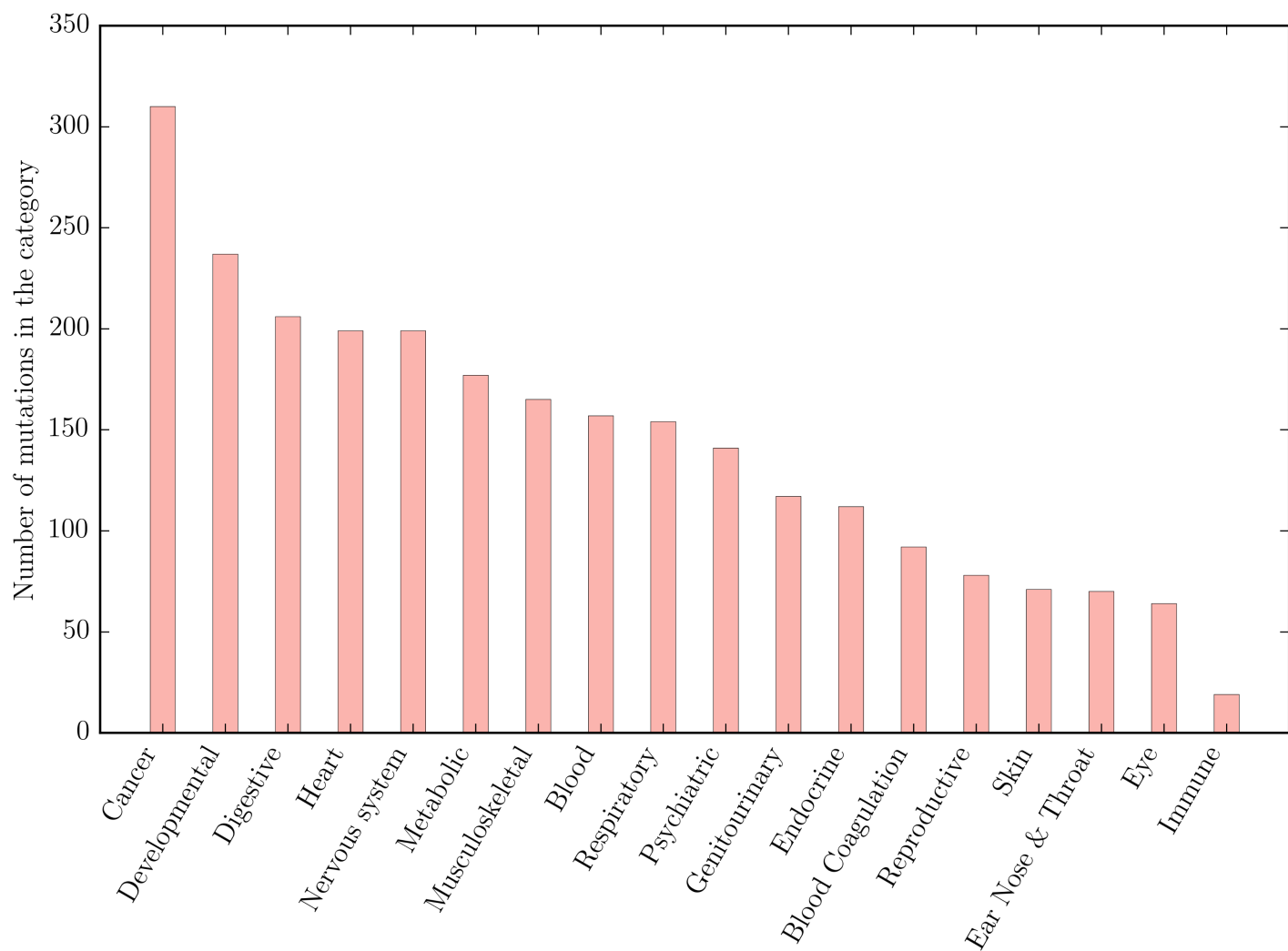

Supplement: Additional file 1: — Supplementary figure: a. Disease group annotation of HGMD coding missense mutations. b. Disease group annotation of HGMD non-coding regulatory mutations. (PDF 1311 kb) [file 13059_2016_1138_MOESM1_ESM.pdf]

**a** **b**

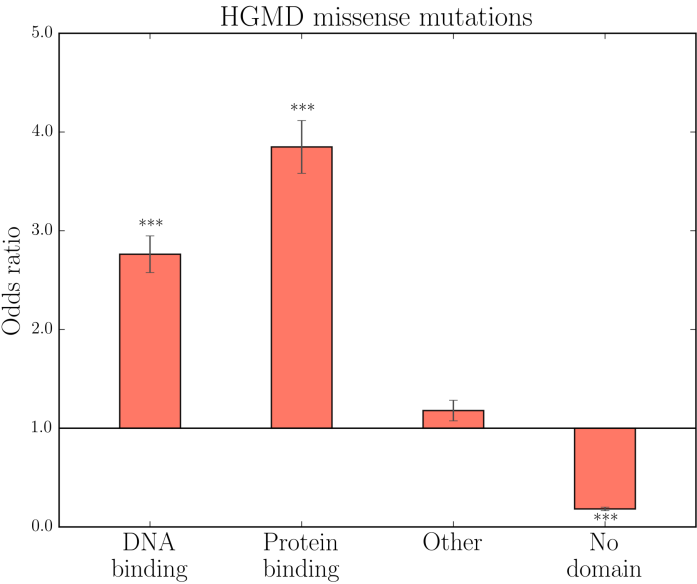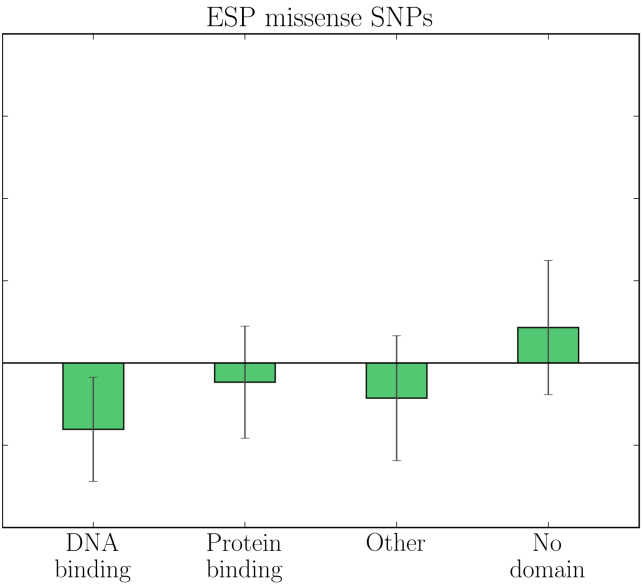

Supplement: Additional file 2: — Supplementary figure: a. Odds ratio for the distribution of transcription factor HGMD missense mutations in different interaction interfaces using only interfaces with co-crystal structures. ***P < 10–3. P values calculated using the Z-test on log odds ratio. Error bars indicate ± standard error (SE). b. Odds ratio for the distribution of transcription factor ESP missense SNPs in different interaction interfaces using only interfaces with co-crystal structures. Error bars indicate ± SE. (PDF 301 kb) [file 13059_2016_1138_MOESM2_ESM.pdf]
